# Biochemical and Molecular Mechanisms of Glucose Uptake Stimulated by Physical Exercise in Insulin Resistance State: Role of Inflammation

**DOI:** 10.5935/abc.20190224

**Published:** 2019-12

**Authors:** Filipe Ferrari, Patrícia Martins Bock, Marcelo Trotte Motta, Lucas Helal

**Affiliations:** 1 Programa de Pós-graduação em Cardiologia e Ciências Cardiovasculares - Faculdade de Medicina - Hospital de Clínicas de Porto Alegre (HCPA) - Universidade Federal do Rio Grande do Sul, Porto Alegre, RS - Brazil; 2 Grupo de Pesquisa em Cardiologia do Exercício - CardioEx (HCPA/UFRGS), Porto Alegre, RS - Brazil; 3 Laboratório de Fisiopatologia do Exercício (LaFiEx), (HCPA/UFRGS), Porto Alegre, RS - Brazil; 4 Instituto de Avaliação de Tecnologias em Saúde (IATS), Hospital de Clínicas de Porto Alegre, Porto Alegre, RS - Brazil; 5 Faculdades Integradas de Taquara, Taquara, RS - Brazil; 6 Departamento de Ciências Biológicas, Universidade Estadual de Feira de Santana (UEFS), Feira de Santana, BA - Brazil

**Keywords:** Exercise, Insulin Resistance, Chronic Inflammation, Glucose Metabolism Disorders, Anti-Inflammatoty Agents, Glucose Transporter Type 4

## Abstract

Obesity associated with systemic inflammation induces insulin resistance (IR), with consequent chronic hyperglycemia. A series of reactions are involved in this process, including increased release of proinflammatory cytokines, and activation of c-Jun N-terminal kinase (JNK), nuclear factor-kappa B (NF-κB) and toll-like receptor 4 (TLR4) receptors. Among the therapeutic tools available nowadays, physical exercise (PE) has a known hypoglycemic effect explained by complex molecular mechanisms, including an increase in insulin receptor phosphorylation, in AMP-activated protein kinase (AMPK) activity, in the Ca2+/calmodulin-dependent protein kinase kinase (CaMKK) pathway, with subsequent activation of peroxisome proliferator-activated receptor gamma coactivator 1-alpha (PGC-1α), Rac1, TBC1 domain family member 1 and 4 (TBC1D1 and TBC1D4), in addition to a variety of signaling molecules, such as GTPases, Rab and soluble N-ethylmaleimide-sensitive factor attached protein receptor (SNARE) proteins. These pathways promote greater translocation of GLUT4 and consequent glucose uptake by the skeletal muscle. Phosphoinositide-dependent kinase (PDK), atypical protein kinase C (aPKC) and some of its isoforms, such as PKC-iota/lambda also seem to play a fundamental role in the transport of glucose. In this sense, the association between autophagy and exercise has also demonstrated a relevant role in the uptake of muscle glucose. Insulin, in turn, uses a phosphoinositide 3-kinase (PI3K)-dependent mechanism, while exercise signal may be triggered by the release of calcium from the sarcoplasmic reticulum. The objective of this review is to describe the main molecular mechanisms of IR and the relationship between PE and glucose uptake.

## Introduction

Insulin resistance (IR) at target tissues is directly related to chronic subclinical inflammation. When inadequately controlled, IR cause a permanent hyperglycemic status, characterizing the pathophysiology of type 2 diabetes mellitus (DM2).^1^ Cardiovascular diseases are the main cause of morbidity and mortality in DM2 patients,^2^ leading to annual costs per year of nearly 40 billion.^3^

Hyperglycemia, *per se*, is a devastating condition for the cardiovascular system. Among the complications caused by chronic hyperglycemia in patients with DM2, there is a reduction in endothelial vasodilator capacity (by reduced nitric oxide availability), increase in advanced glycation end products, in addition to increased oxidative stress, which leads to endothelial dysfunction and atherogenesis in long term, and increased cardiovascular risk.^4,5^

Physical exercise (PE), combined with pharmacologic therapy, is an effective strategy in the approach of DM2 patients, with direct effect on glycemic ontrol,^6,7^ due to its capacity in reducing blood glucose concentrations^8^ and its anti-inflammatory effect in long term,^9^ with potential positive effect in reducing cardiovascular complications in these patients.

Muscle contraction acutely increases trigger biochemical reactions that culminate in increased glucose uptake by the muscle. This is caused by two important mechanisms - increase in insulin sensitivity^10^ and translocation of the type 4 glucose transporter (GLUT4) to the cell surface independent of insulin use.^11^ In addition, PE chronically increases intramuscular GLUT4 content^12^ and reduces the inflammatory state, especially by the release of anti-inflammatory cytokines^13^ and reduction in total lipid content.^14^

The objective of this review is to provide an overview of the regulation of glucose uptake in IR and chronic subclinical inflammation, and the role of PE in this situation. First, we present a discussion about biochemical and molecular mechanisms of the hypoglycemic effect of PE, with special attention to the increase in insulin sensitivity and translocation of de GLUT4 independent of insulin; then, we present evidence of the role of PE as an anti-inflammatory strategy and its association with IR.

### Signaling of insulin and glucose uptake by skeletal muscle

Insulin is a peptide hormone released by the pancreas, specifically by beta cells of the pancreatic islets.^15^ Intracellular signaling of insulin in insulin-sensitive tissues requires binding of the hormone to a specific membrane receptor, named insulin receptor, composed by four subunits: two α subunits located in the external part of the membrane, and two transmembrane, β subunits. Insulin binds to the α subunits, and activate the kinase activity of beta subunits, which promotes the self-phosphorylation of tyrosine residues in the intracellular region of insulin receptor.^16^ This generates the recruitment of adaptor proteins and phosphorylation of several protein substrate, including members of the insulin receptor substrate family - IRS-1, 2, 3 and 4.^17^ Among these members, phosphorylation of IRS-1 and IRS-2 into tyrosine - by addition of a phosphate group - bind to and activate Src homology-2 (SH2) domains, such as the phosphoinositide 3-kinase (PI3K). The SH2 domain exhibits approximately 100 amino acids and is able to recognize and bind to phosphorylated tyrosine.^18^ PI3K, in turn, catalyzes the formation of phosphatidylinositol (3,4,5)-trisphosphate (PI3P),^19^ an allosteric regulator of phosphoinositide-dependent kinase (PDK).^20^ PDK activates one of the isoforms of protein kinase B (PKB), also known as Akt, and the atypical protein kinase C (aPKC).^21^ There is evidence that aPKC is essential for insulin-stimulated glucose transport in skeletal muscle; its activation seems to be compromised in IR,^22^ and potentialized by PE.^23^ Among the aPKC isoforms, the aPKC lambda/iota has shown an important role in glucose transport. This enzyme phosphorylates the double C2-like domain-containing protein (DOC2b), which regulates the soluble N-ethylmaleimide-sensitive factor attached protein receptor (SNARE), facilitating the interaction with syntaxin-4 and promoting the fusion of GLUT4-containing vesicles with the plasma membrane.^24^ In addition to aPKC, other PKC isoforms are also involved in GLUT4 translocation, including PKCα and PKCθ, which are activated by the increase in intracellular calcium.^25^

Besides the PKC isoforms, the Akt enzyme promotes the phosphorilation of the Rab GTPase-activating proteins (RabGAPs), that involve the TBC1 domain family member 4 (TBC1D4) and TBC1 domain family member 1 (TBC1D1). This enables the dissociation of the Rab protein, and consequently, increased uptake of glucose by increased GLUT4 translocation.^26^ The TBC1D1 and the TBC1D4 proteins act cooperatively regulating the translocation of GLUT4 in response to a stimulus, since both are co-expressed in skeletal muscle.^27^ In summary, TBC1D4, previously known as Akt substrate of 160 kDa (AS160), is a protein that, when phosphorylated into treonin-642, helps in the translocation of GLUT4-containing vesicles to the membrane, in GLUT4 expression, leading to increased glucose uptake.^28^ Akt also induces the phosphorylation of serine/threonine kinase with an atypical placement of the catalytic lysine, called with-no-lysine kinase (WNK1), with omnipresent expression, including the skeletal muscle. WNK1, in turn, phosphorylates the TBC1D4 enzyme, promoting the translocation of GLUT4 in the skeletal muscle.^29^

Therefore, activation of the cascade that involves PI3K/Akt enzymes allows the entry of glucose into cells by facilitated diffusion, by stimulation of translocation of GLUT4 from intracellular vesicles to the plasma membrane.^30^ In addition to GLUT4 translocation, PI3K simultaneously stimulates the synthesis of hepatic and muscle glycogen.^31^ In this context, another important mechanism was proposed. Previous studies using cell cultures have shown that inhibition of the endogenous Rac1 (member of the Rho-family of GTPases) blocked the insulin-induced GLUT4 translocation.^32,33^ Rac1, in turn, was described as essential in the stimulation of insulin-mediated glucose uptake in skeletal muscle and glucose homeostasis in the whole body,^34,35^ exerting a preponderant role in the regulation of insulin-induced GLUT4 translocation, as observed in cultured muscle cells.^36^

Also, when endogenous production of insulin is compromised (or in state of very high insulin resistance), the role of PE is even more important due to its insulin-independent hypoglycemic effect.^37^

### Physical exercise in the regulation of glucose uptake in skeletal muscle

During PE, the utilization of energy substrates (mainly glucose and free fatty acids) considerably increases in relation to rest. These substrates originate from intramuscular stores, hepatic production and fat tissue mobilization by hormone-sensitive lipase.^38^

Both acute aerobic exercise and chronic exercise training can potentiate the action of insulin, and evidence from animal models has helped us to understand the mechanisms involved. In rats fed a high-fat diet, acute PE seems to affect the activation of insulin receptor, since a unique session of exercise increases insulin-stimulated IR phosphorylation in skeletal muscles.^39^ In obese rats, both high-volume exercise (six-hour duration) and low-volume exercise (45 minutes) were effective in increasing insulin sensitivity, by increased phosphorylation of IR, IRS-1 and Akt.^40^ Another experiment with rats showed an improvement in insulin sensitivity in adipocytes after seven weeks of daily aerobic exercise (60-minute duration), mediated by increased tyrosine phosphorylation in IRS-1 and IRS-2 and greater association of IRS-1 with PI3K and, consequently, increased phosphorylation of Akt protein.^41^

In addition, PE can increase glucose uptake in the muscle by other pathways that involve a key enzyme activated by muscle contraction, named AMP-activated protein kinase (AMPK). AMPK is a heterotrimeric molecule composed of a catalytic subunit (alpha) and two regulatory subunits (beta and gamma), with the following isoforms β1, β2, γ1, γ2 and γ3. It is activated by phosphorylation of a threonine-172 residue within the activation loop of the α subunit.^42^ The activation of AMPK can result from an energy imbalance caused by muscle contraction.^43^ Among the proteins that regulate AMPK, liver kinase B1 (LKB1) is currently considered the main protein involved in AMPK phosphorylation.^44^ The activation of AMPK and LKB1 during exercise has been widely demonstrated in animals and humans.^43,45^

It is worth pointing out AMPK-stimulated glucose transport seems to be mediated by multiple factors - by increase of intracellular concentrations of Ca^++^ and bradykinin (plasma polypeptide that causes vasodilation), increased activity of endothelial nitric oxide synthase (which increases vasodilation and the availability of nitric oxide), by activation of mitogen-activated protein kinase (MAPK), activation of Ca^(2+)^ /calmodulin-dependent protein kinase (CaMK), activation of protein kinase C (PKC), and even hypoxia.^46,47^ All these factors are necessary for an effective translocation of GLUT4 and consequent entry of glucose into the cells.

In addition, there is evidence suggesting that activation of AMPK in skeletal muscle can increase lipid oxidation, and thereby glycogen resynthesis can adapt to PE (by sparing muscle glycogen) by stimulation of muscle contraction.^48^ Some myokines, including interleukin-15 (IL-15) and interleukin-6 (IL-6), increase the expression of GLUT4 in adipose tissue, which can potentiate PE-induced glucose uptake,^49^ and also activate AMPK and GLUT4 translocation to the cell surface.^50^ Activation of AMPK is also important since as it promotes the phosphorylation of TBC1D1 and TBC1D4. Studies have shown that both acute and chronic exercise increase the expression of AMPK, TBC1D1, TBC1D4 and GLUT4 in skeletal muscles in humans.^51,52^ It was also reported that in contracted epitrochlearis muscles of rats, TBC1D4 phosphorylation was increased, and this effect persisted for 3-4 hours after the animals swam for four 30-min bouts with a 5-min rest between bouts.^53^ Kjøbsted et al.^54^ corroborated this hypothesis in a recent study showing that increased phosphorylation of TBC1D4 stimulated by insulin in exercised muscles improves insulin sensitivity.

Another important event associated with PE and AMPK activation is the activation of the of peroxisome proliferator-activated receptor gamma coactivator 1-alpha (PGC-1α),^55^ mediated p38 MAPK and histone deacetylase-5 (HDAC5).^56^ In addition, phosphorylation of Ca2+/calmodulin-dependent protein kinase (CaMKK) followed by activation of PGC-1α, can be induced by low-intensity, resisted exercise, suggesting that PE-induced GLUT4 translocation can be achieved by several modalities.^57^ On the other hand, other important proteins, as the case of Pac1,^34,35^ do not require activation of the AMPK pathway to promote PE-induced glucose uptake in skeletal muscle.^34,35^

Studies have indicated that muscle elongation contributes to activation of Rac1.^58,59^ Silow et al.^58^ have shown that Rac1 signaling is impaired in muscles resistant to insulin in rats and humans. The importance of Rac1 in this context is attributed to its effects on actin cytoskeleton. Thus, dysregulation of Rac1 and actin cytoskeleton in the skeletal muscle can be new molecular candidates that contribute to the phenotype of IR and DM2.^58^ More recent data have supported these findings, suggesting that Rac1 essentially contributes to PE-stimulated glucose uptake.^60,61^ However, it is important to mention that previous studies have shown that short exercise completely restored insulin sensitivity in Rac1-deficient muscle containing RI.^62^ Therefore, although Rac1 is essential for regulation of glucose transport stimulated by PE, it is dispensable for the insulin sensitizing effect of exercise. This is important since Rac1 is dysfunctional in insulin-resistant muscle.^63^ These findings indicate that other pathways different from the Rac1 pathway, can exhibit more pronounced effects of insulin sensitization during PE.^64^

**Figure 1:**
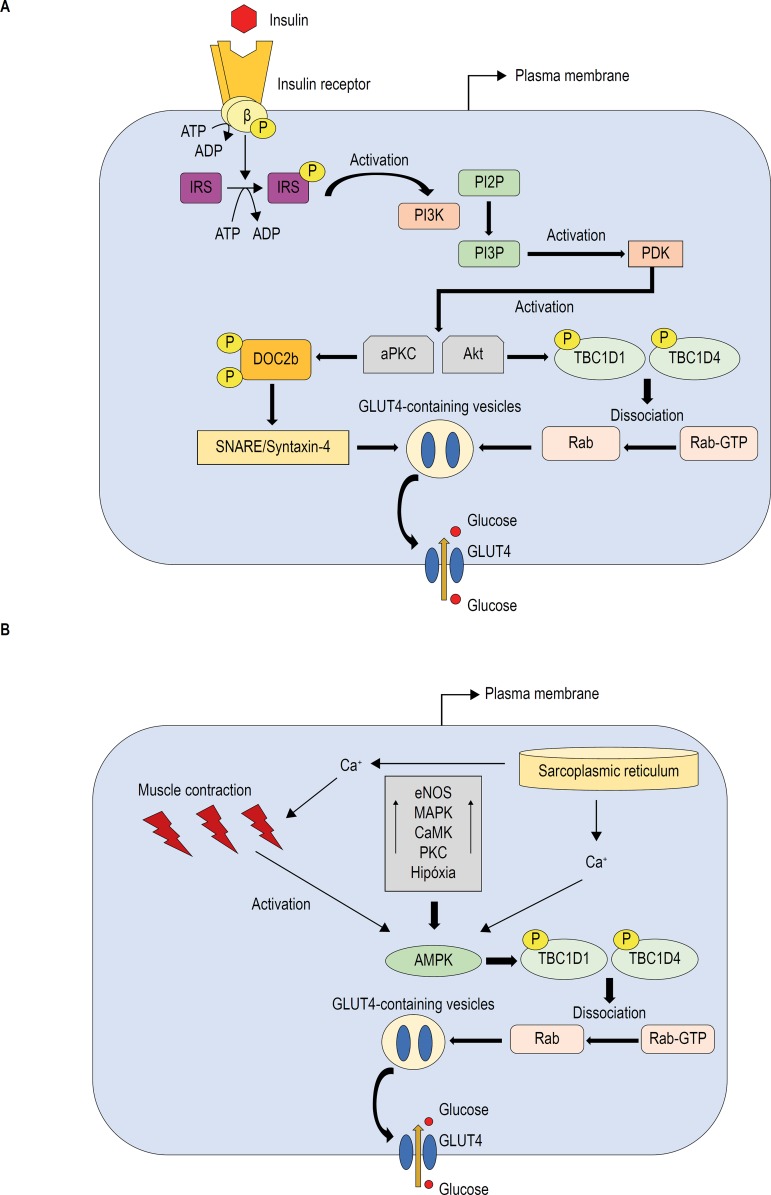
Schematic representation of the main pathways that promote the translocation of GLUT4-containing vesicles to the membrane in the skeletal muscle induced by insulin (A) and insulin-independent pathways during physical exercise (B) P: Phosphorylation; ATP: Adenosine triphosphate; ADP: Adenosine diphosphate; IRS: insulin receptor substrate; PI3K: phosphoinositide 3-kinase; PI2P: phosphatidylinositol-4,5-bisphosphate; PI3P: phosphatidylinositol (3,4,5)-trisphosphate; PDK: phosphoinositidedependent kinase; aPKC: atypical protein kinase C; DOC2b: double C2-like domain-containing protein; SNARE: soluble N-ethylmaleimide-sensitive factor attached protein receptor; TBC1D1: TBC1 domain family member 1; TBC1D4: TBC1 domain family member 4; GLUT4: glucose transporter type;Ca+: Calcium; eNOS: nitric oxide synthase; MAPK: mitogen-activated protein kinase; CaMK: Ca2+/calmodulin-dependent protein kinase; PKC: protein kinase C; AMPK: AMP-activated protein kinase.

A schematic illustration of GLUT4 translocation mediated by insulin and by muscle contraction is presented in Figure 1.

Other important and complex mechanisms related to the AMPK pathway need to be mentioned. For example, its relationship with autophagy, a process involved with glucose metabolism and insulin sensitivity. Autophagy is a self-degradative process that occurs via lysossomal pathway that plays a role in the removal of malformed or aggregated proteins, eliminating damaged organelles, similarly to mitochondria and sarcoplasmic reticulum. Autophagy is generally considered a survival mechanism, although its dysregulation has been associated with non-apoptotic cell death.^65,66^

The relationship between autophagy, PE and metabolic regulation is still a little explored area. However, there is increasing evidence that the autophagic process is strongly induced during physical training,^67,68^ and seems to play an important role in the metabolism of skeletal muscle.^69^ In this regard, autophagy can regulate the homeostasis of muscle glucose and contribute to the reduction of RI in response to PE.^70^ These data are corroborated by He et al.,^71^ in an experiment conducted with mice, showing that mice with induced allelic loss of *Beclin 1*, an autophagy-related gene that promotes a decrease in autophagy in the skeletal muscle, had impaired exercise-induced GLUT4 plasma membrane localization. These data suggest an important role of autophagy and *Beclin 1* in improving glucose uptake in response to PE. For example, a single bout of running for 90 minutes on a treadmill was sufficient to induce autophagy in the skeletal muscle and in the brain of mice.^68^ One of the hypotheses that may explain the mechanisms involved in this scenario is that PE can increase the concentrations of proteins of the sestrins (SESNs) induced by stress, such as SESN1 and SESN3, which not only increase the autophagic activity, but also interact with AMPK, and stimulate its activation.^72,73^ The induction of SESNs inhibits the mechanistic target of rapamycin complex 1 (mTORC1) activity by stimulation of AMPK.^73^ Thus, the interaction between sestrin and AMPK induced by PE may be involved in the beneficial metabolic effect of training, activating autophagy. This interaction provides a molecular mechanism that is a potential target in metabolic syndromes.

### Obesity, inflammation and insulin resistance

IR develops silently and may lead to pancreatic failure, starting with a resistance to insulin activity in the target-tissues, followed by an increase in pancreatic insulin production in response to such IR, and ultimately with incapacity of the pancreas to continue insulin production. This fact opens the door to DM2, characterized by an acquired chronic hyperglycemia associated with other diseases including hypertension and dyslipidemia. The main factors that cause this syndrome are obesity, sedentary lifestyle and genetic factors.^74^ IR is characterized by pathological changes in several steps of insulin metabolic pathway,^75^ with simultaneous increase in endogenous production of hepatic glucose, leading to chronic hyperglycemia.^76^ Today, obesity, especially visceral obesity, is recognized as one of the main risk factors of IR.^77^

Several mechanisms are involved in the etiopathogenesis of obesity-related IR, characterized by changes in several steps of insulin signaling, with reduction in IR concentration and kinase activity, in IRS-1 and IRS-2^78^ phosphorylation into tyrosine, and in PI3K activity.^79^ In addition, a significant increase in abdominal adipose tissue induces the delivery of free fatty to the liver through the portal vein, aggravating hepatic insulin resistance,^80^ thereby increasing the release of proinflammatory cytokines through the portal vein, which acts as a feedback to the process.^81^

The role of chronic inflammation in this scenario cannot be excluded. IR is related to obesity-induced inflammation, process already described in the 90’s. In this decade, several studies evaluated the association of IR with traditional inflammatory markers, such as tumor necrosis factor-alpha (TNF-α) and showed that adipocytes treated with TNF-α had impaired insulin signaling. This response was associated with reduced IRS-1 and GLUT-482 transcription.^82^

Pro-inflammatory cytokines, such as the TNF-α, can lead to activation of c-Jun N-terminal kinase (JNK), a critical enzyme in inflammation associated with obesity and IR,^83^ by activating serine or threonine kinase, thereby reducing insulin signaling by phosphorylation of proteins into serine or threonine residues.^84^ Besides, activation of this enzyme is related with signaling pathways that activate nuclear factor-kappa B (NF-B) which, in turn, stimulates the production of pro-inflammatory cytokines.^85^ The activation of JNK also promotes NF-B activation in pancreatic islets, and therefore, perpetuating a vicious cycle of β-cells dysfunction induced by inflammation, which in turn aggravates the chronic inflammatory process.^86^ This feedback causes more macrophage recruitment, which together with hypertrophic adipocytes, release more pro-inflammatory cytokines.^87^

Additionally, circulating free fatty acids, as well as other ligands such as bacterial lipopolysaccharides, are able to activate transmembrane proteins known as toll-like receptor 4 (TLR-4), that trigger inflammatory pathways, reducing glucose uptake by insulin signaling^88^ in a process called metabolic inflammation.^89^ TLR-4 is ubiquitously expressed throughout the cells, including the adipose tissue. In the development of obesity, there is greater infiltration of immune cells in this tissue, particularly macrophages, which show increased expression of TLR4.^90^ Free fatty acids bind to TLR-4, activating JNK and IκB kinase (IKK).^91^ Because IRS-1 are target of both enzymes, this process affects tyrosine phosphorylation, resulting in reduced GLUT4 translocation.^92^

**Figure 2:**
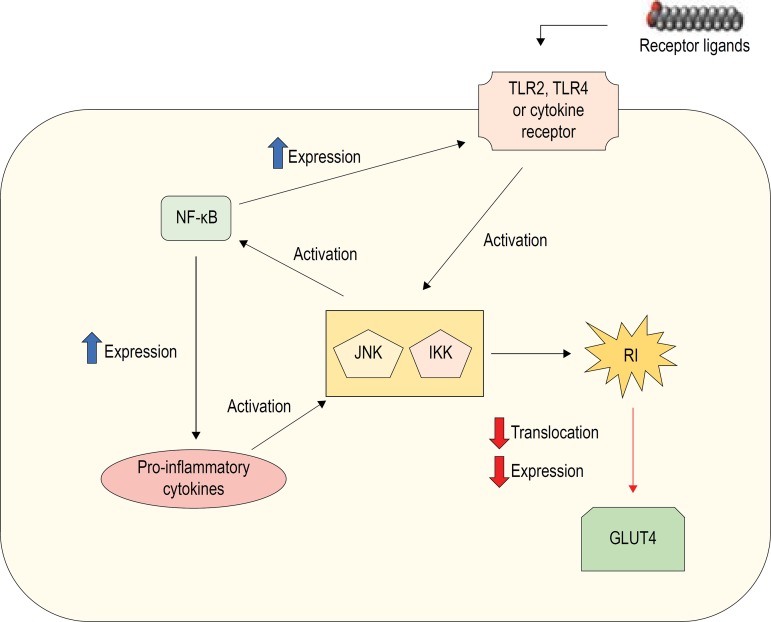
Schematic representation of activation of TLR 2, TLR4 or cytokine receptor by extracellular ligands and induction of inflammation and insulin resistance in an adipocyte. TLR2: toll-like receptor 2; TLR4: toll-like receptor 4; NF-κB: nuclear factor-kappa β; JNK: c-Jun N-terminal kinase; IKK: IkB kinase; GLUT4: glucose transporter type 4; IR: insulin resistance.

Activation of IKK causes phosphorylation and subsequent proteasomal degradation of IKKβ, inducing activation of NF-κB. Degradation of IKKβ triggers the gene transcription of inflammatory mediators, such as TNF-α and interleukin-6 (IL-6).^93^ Also, IKKβ promotes serine phosphorylation of insulin receptor and IRS-1 and IRS-2 substrates, which reduces insulin signaling in different tissues.^94^ These processes are schematically illustrated in Figure 2.

In summary, the increase in circulating free fatty acids is a metabolic characteristic of insulin-resistant state, which may cause IR by several mechanisms. Evidence has suggested that excess adipose tissue reduces insulin receptor phosphorylation and promotes chronic activation of pro-inflammatory cytokines and circulating fatty acids, which may lead to deterioration of the tissue response to insulin. Adipose tissue, previously believed to be a mere place of energy storage, has shown to be an important endocrine and pro-inflammatory organ. It is more evident with visceral white adipose tissue that exhibits macrophage infiltration with local production of interleukins, which can help in the development of local and systemic IR.^95-97^ Therefore, strategies targeting anti-inflammatory responses in the adipose tissue, such as PE, may have beneficial effects on individual’s health status, alleviating the burden of obesity in endocrine dysregulation.

### Physical exercise in obesity and insulin resistance

The beneficial role of PE has been increasingly recognized in increasing insulin sensitivity, independent of body fat reduction by the training.^98^ The protective effect of PE may be attributed to the anti-inflammatory effect of physical training mediated by a reduction in visceral fat and/or induction of an inflammatory environment, with elevation in IL-10 and interleukin-1 receptor antagonist (IL-1Ra) concentrations, and reduction in IL-6 and TNF-α.^99^

As previously mentioned, visceral obesity is an important factor for the development of DM, which may be related to the increase in IL-6 and TNF-α.^100^ Regular exercise can reduce baseline production of IL-6, by decreasing its plasma concentration at rest.^101^ After acute moderate-intensity exercise, plasma IL-6 can increase in up to 100 times after a marathon (even though this is not adequate for obese individuals), but rapidly decreases compared with pre-exercise values.^101^ This cytokines also stimulates proliferation of β-cells, and increased IL-6 concentrations in response to PE can stimulate the release of glucagon-like peptide-1 (GLP-1), an important hormone that stimulates insulin secretion.^102,103^ These evidences support a beneficial effect of IL-6 in the regulation of insulin secretion, which undoubtedly contributes to DM prevention.

Regarding AMPK in DM2 and IR scenario, many studies have suggested that muscle contraction plays a central role, regardless of insulinemic status, where the activity of AMPK-α2 in skeletal muscle in response to PE was similar to that in individuals without DM2, indicating a normal functioning of muscle AMPK in diabetics, which is particularly important in IR conditions.^104^ In another study, an acute bout of aerobic exercise (one hour duration) at 75% of VO_2_max did not increase insulin sensitivity in obese diabetic subjects. Nevertheless, after seven sessions, there was an increment in glucose uptake rate, possibly stimulated by increased AMPK activity. It is of note that no difference was observed in the expression of proteins of insulin signaling pathways post-exercise compared with baseline.^105^

The action of Akt protein, previously mentioned as an important mediator of GLUT4 mobilization from GLUT4-containing vesicles to the membrane, may be impaired by the mammalian homolog of Ddosophila tribbles TRB3, whose expression is increased in obesity.^106^ However, PE seems to be able to reduce the expression of this protein TRB3. A study showed that acute exercise reduced TRB3 expression and reversed Akt phosphorylation in the skeletal muscle of obese animals.^107^ On the other hand, one session of swimming reduced TRB3 levels in the hypothalamus of obese rats.^108^ In a recent study by Wang et al.,^109^ the authors showed that aerobic training contributed to reduce inflammatory factors in mice with induced DM2. In addition to reducing body weight, there was a inhibition of TLR4 in hepatic cells of these animals, which, in turn, increased AMPK expression, ultimately contributing to the improvement of inflammation and IR.^109^ Therefore, this pathway would also explain the importance of aerobic exercise in improving insulin sensitivity and glycemic control in DM2. These findings may lead to further studies, especially in humans, and open new horizons for the treatment of obesity and IR.

PE can also exert beneficial effects on cardiovascular system by mechanisms including the increase in adiponectin.^110^ Among its several functions, adiponectin can greatly suppress hepatic glucogenesis, stimulating the oxidation of fatty acids in the skeletal muscle and inhibiting the transcription of genes involved in glucose production. In insulin-responsive tissues, adiponectin improves the sensitivity to this hormone.^111,112^ Hypoadiponectinemia, defined by plasma adiponectin levels lower than 4.0 µg/mL, was associated with decreased levels of circulating high-density lipoprotein, triglycerides and glucose, and increased risk of metabolic syndrome. Also, the risk for atherosclerosis was twice as high in individuals with low adiponectin levels.^113^

The improvement in adiponectin levels has been associated with loss of subcutaneous and visceral adipose tissue induced by PE.^114^ Studies have shown that aerobic PE alone^115^ or combined with diet^116^ significantly increase adiponectin levels in adipose tissue in obese subjects, regardless of changes in body composition. In addition, PE, particularly aerobic exercise, was able to change the body fat distribution, by reduction of pro-inflammatory cytokines and improvement of insulin sensitivity.^112^

Finally, plasma levels of resistin (protein related to IR and glucose intolerance), decreased after PE programs.^117,118^ Resistin is commonly found in obese individuals, and seems to be involved in IR.^119^ It was recently demonstrated that accumulation of this protein is associated with lower survival of DM2 patients, and concentrations above 11 ng/mL indicate increased risk in these patients.^120^ Reduction in resistin concentrations by interventions, such as PE, may be related to reduction in inflammation via release of anti-inflammatory cytokines rather than changes in glucose metabolism and reductions of body mass.^121^

Therefore, obesity, in consonance with inflammatory process, can contribute to the increase in important inflammatory markers, such as pro-inflammatory cytokines. Available evidence has indicated that PE reduces these markers, regardless of a reduction in body weight.

## Final considerations

PE stimulates many complex molecular and biochemical mechanisms, which promote a substantial improvement in insulin signaling and glucose uptake in IR states. It is important to highlight that evidences for the role of PE in reduction of the inflammatory process in IR associated with obesity were also presented.
